# Stroke as an unusual initial presentation of ‘malignant’ middle cerebral artery infarction involvement in systemic lupus erythematosus

**DOI:** 10.1097/MS9.0000000000001502

**Published:** 2023-11-16

**Authors:** Yousef Mahmoud Nimer Habes, Manar Ahmaro, Yasmin Dwikat, Afnan W. M. Jobran, Fawzy Mazen Abunejma, Mohammed Abdulrazzak

**Affiliations:** a Faculty of Medicine, Al Quds University, Jerusalem; b Internal Medicine, Istishari Arab Hospital, Ramallah; c Pediatric Rheumatology Unit, Al-Ahli Hospital, Hebron, Palestine; d Faculty of Medicine, University of Aleppo, Aleppo, Syria

**Keywords:** autoimmune disease, case report, middle cerebral artery, stroke, systemic lupus erythematosus

## Abstract

**Introduction and importance:**

SLE, or systemic lupus erythematosus, is a chronic autoimmune condition of uncertain origin characterized by the presence of autoantibodies that target the body’s own antigens.

**Case presentation:**

A 16-year-old female presented at the emergency room with a right-sided lip droop and subsequently developed symptoms consistent with a malignant hemispheric infarction, including altered consciousness, hemiplegia, and forced gaze deviation. Her laboratory results were within the normal range. However, a brain MRI revealed cerebral edema and a massive infarction in the middle cerebral artery (MCA) region. Subsequently, her serologic profile was indicative of SLE, leading to a later diagnosis.

**Clinical discussion:**

The patient in this case presented with symptoms suggestive of a stroke. A CT scan showed MCA occlusion, leading to a diagnosis of malignant MCA syndrome. The patient was also diagnosed with systemic lupus erythematosus, which is associated with an increased risk of stroke. Inflammation-induced thrombosis and CNS vasculitis are potential mechanisms linking SLE and stroke.

**Conclusion:**

This case serves as an example of a sudden and potentially life-threatening presentation of SLE, underscoring the importance of early detection and targeted treatment that can influence the course of the disease.

## Introduction

Highlights

- The case presents an important clinical scenario of stroke as the initial manifestation of systemic lupus erythematosus (SLE) in a young patient.
- SLE increasing the risk of stroke, especially in young patients is an important clinical correlation to be recognized.
- This case serves as an example of a sudden and potentially life-threatening presentation of SLE.

SLE, or systemic lupus erythematosus, is a chronic autoimmune condition of uncertain origin characterized by the presence of autoantibodies that target the body’s own antigens, primarily antinuclear antibodies. This immune response leads to inflammation-mediated damage to multiple organs throughout the body, potentially affecting any organ system. Notably, the nervous system is susceptible to involvement in SLE, giving rise to a wide array of neurological and psychiatric symptoms^1^.

Neurological manifestations are a recognized aspect of SLE and represent a significant contributor to primary mortality. For instance, a decade-long cohort study involving 1000 SLE patients revealed that 12% of mortality in this population was attributed to cerebrovascular events^1^. Additionally, research indicates that the central nervous system (CNS) experiences involvement in a range of 10–80% of patients either prior to their diagnosis or during the course of their illness^2^. One of the manifestations associated with neuropsychiatric SLE (NPSLE) is stroke, which occurs in ~19% of patients, with most cases developing within the first 5 years after diagnosis^3^.

In this report, we present a unique case in which two uncommon conditions co-occurred in the same patient. A severe stroke in the middle cerebral artery (MCA) territory, known as a malignant MCA stroke. This malignant hemispheric infarction served as the initial manifestation of her underlying SLE. This case underscores the critical importance of considering autoimmune diseases like SLE when evaluating patients with stroke and transient ischemic attack symptoms, particularly in young individuals with no conventional risk factors.

## Case presentation

A 16-year-old female arrived at the emergency room with sudden right-sided mouth deviation, slurred speech, vision loss, dizziness, and confusion. Her family noted one instance of vomiting and jerky movement in her left lower limb, with no prior history of similar episodes. Her medical history was unremarkable, except for persistent tension-like headaches over the past few months, which had been investigated with electroencephalogram and computed tomography (CT) scans 2 months earlier, revealing no abnormalities. Prior to admission, the patient had experienced gastroenteritis a week ago. She also reported unintentional weight loss of 4 kg over several months, metrorrhagia, one episode of syncope, one episode of exertional dyspnea with chest pain one day prior to admission, two days of unilateral knee pain, and an unmeasured fever for 3 days prior to admission. She also complained of frequent fatigue but denied any skin issues, behavioral changes, or previous seizures.

Upon admission, the patient was confused but had stable vital signs: a temperature of 36.5°C, a pulse of 80 beats per minute, a respiratory rate of 15 breaths per minute, and a blood pressure of 107/75 mmHg. Physical examinations of the heart, lungs, and abdomen showed no abnormalities. Neurologically, she displayed lethargy but could be roused, with fluctuating attention and concentration levels. She exhibited right-sided mouth deviation, slurred speech, and hemiparesis affecting the left side of her face and upper and lower limbs, along with numbness in the same distribution. Cranial nerves were intact, except for deconjugate gaze and diplopia, and the Babinski sign was positive. There were no signs of meningitis.

The patient had no history of medication use, allergies, or surgical procedures. Initial lab tests, including complete blood count, serum electrolytes, biochemistry, lipid profile, liver function tests, and kidney function tests, all returned within normal ranges. Serum albumin was slightly decreased at 3.3 mEq/l. Coagulation tests showed a prolonged prothrombin time of 17.4 s and a high international normalized ratio of 1.51. Initially, encephalitis was strongly suspected but later ruled out through a cerebrospinal fluid analysis and additional lab tests. Prolactin levels were elevated at 37.1 ng/ml, suggesting a true seizure, while blood, and urine cultures were negative.

Additional investigations, including a chest radiographs, electrocardiography, and echocardiogram, revealed normal results, ruling out cardiac embolism as the cause. The serology for Hepatitis B Virus, Epstein–Barr virus (EBV), and cytomegalovirus (CMV) came back negative.

Autoantibody testing showed highly positive antinuclear antibodies (ANA) and anti-double stranded DNA, while other antibodies were negative. Inflammatory markers were elevated, with an erythrocyte sedimentation rate (ESR) of 68 mm/h and a C-Reactive protein (CRP) of 8.6 mg/l. Complement levels (C3, C4) were within the normal range. Genetic studies were also conducted, revealing unconventional findings related to hereditary thrombophilia.

Subsequently, the patient received a diagnosis of SLE based on the European League Against Rheumatism/American College of Rheumatology (EULAR/ACR) 2019 criteria and was initiated on a treatment regimen involving steroids and azathioprine.

**Figure 1:**
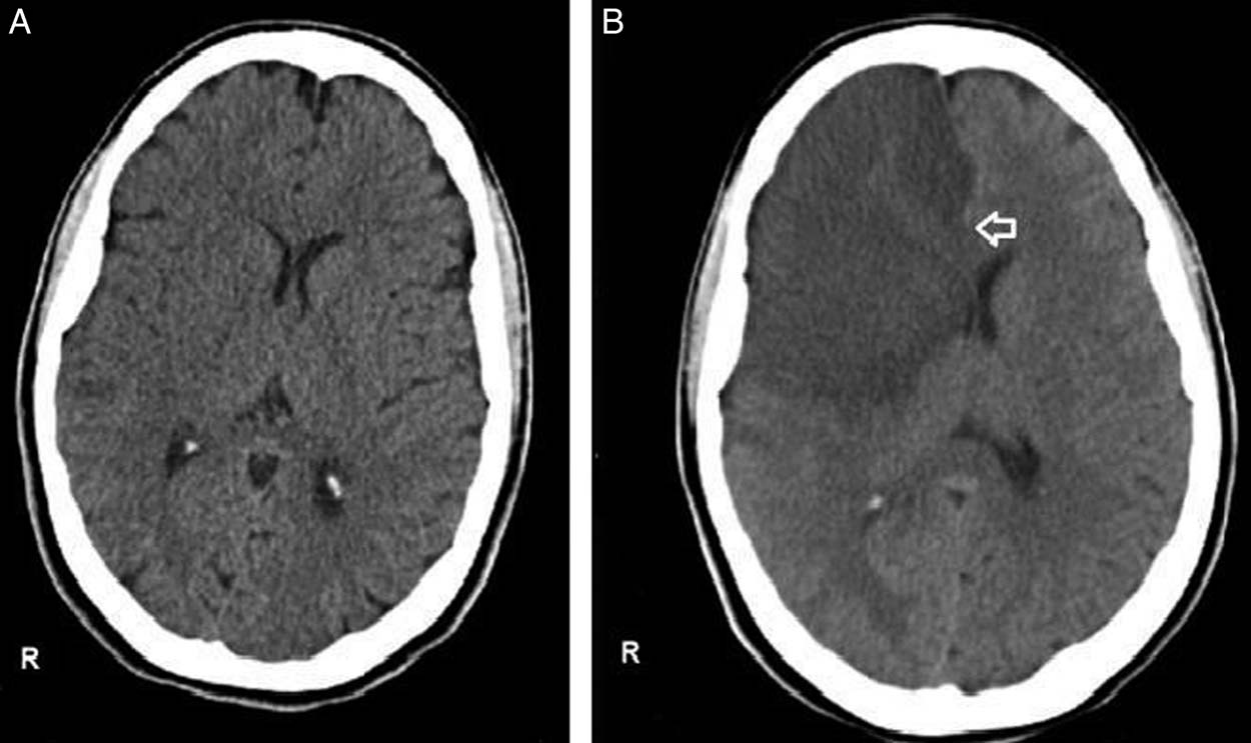
(A) An initial axial head CT scan without contrast upon admission revealed no discernible pathological abnormalities. (B) A subsequent head CT scan without contrast, conducted on the following day, displayed a substantial hypodense area in the right frontal-parieto-temporal lobes, leading to a pronounced mass effect. This was characterized by the loss of distinction between gray and white matter, obliteration of the underlying sulci, compression of the right lateral ventricle, and a substantial 11 mm midline shift toward the left side.

**Figure 2:**
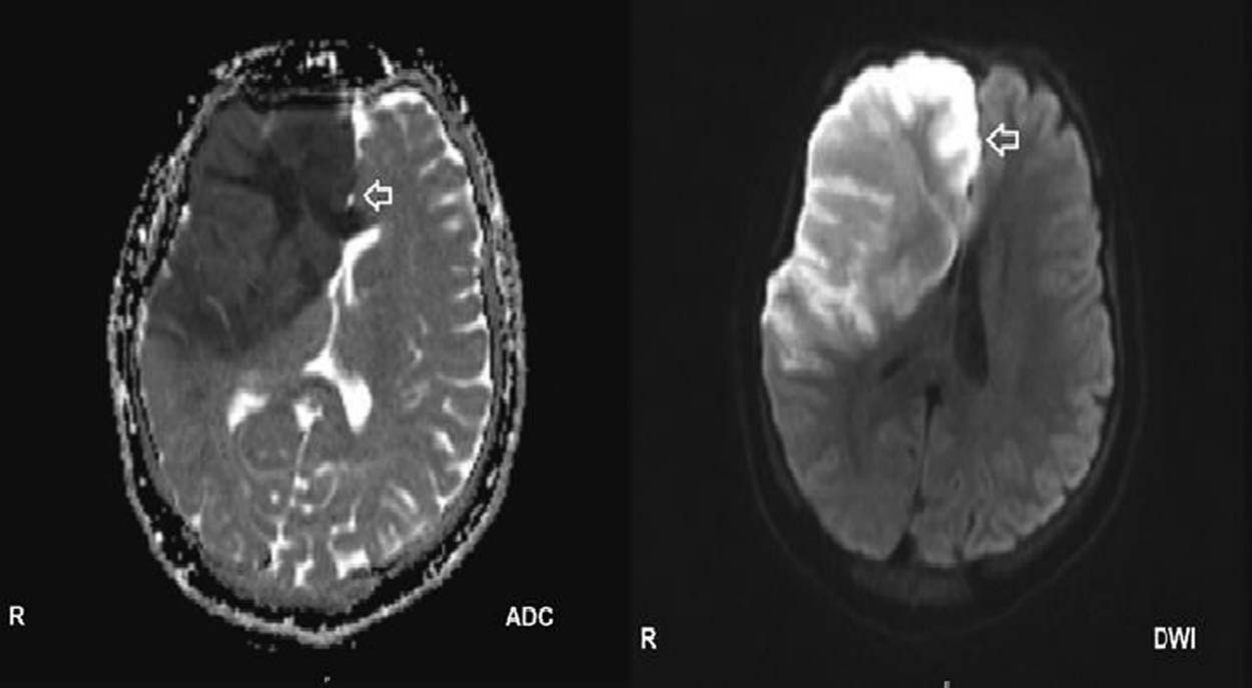
An axial head MRI utilizing diffuse weighted imaging (DWI) reveals a significant area of hyperintensity in the fronto-parieto-temporal lobes, along with hypo-intensity observed on the ADC map. This pattern is highly indicative of neuronal death and is commonly associated with acute infarction in the right MCA (middle cerebral artery) and ACA (anterior cerebral artery) regions.

**Figure 3:**
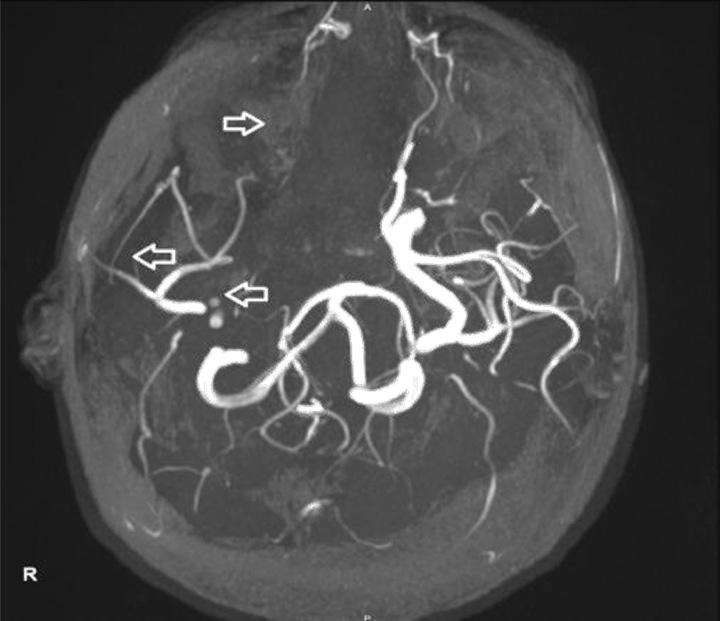
The MRA image displays occlusion of the right anterior cerebral and middle cerebral arteries (indicated by arrows) resulting from the complete blockage of the intracranial supra-clinoid segment of the right internal carotid artery (ICA).

An initial head CT scan showed no pathological findings (Fig. 1), but due to clinical deterioration and decreased consciousness, a repeated CT scan was performed along with an MRI (Fig. 2). The MRI revealed a massive infarction in the MCA region, confirmed by MRA and CT angiogram (Fig. 3), with evidence of cerebral edema. These findings were consistent with malignant cerebral hemispheric infarction, also known as malignant MCA syndrome.

A decision was made to perform an urgent decompressive fronto-parieto-temporal craniectomy within 48 h of her presentation to alleviate intracranial pressure and enhance cerebral blood perfusion. The surgery proceeded without complications, and she received 2 units of packed red blood cells and 1 unit of fresh frozen plasma. She exhibited clinical improvement and was subsequently discharged with a scheduled follow-up.

## Discussion

The patient’s initial presentation included right-sided mouth deviation, vision loss, slurred speech, confusion, dizziness, vomiting, and abnormal movements in the left lower limb. Furthermore, she exhibited hemiparesis affecting the left side of her face and upper and lower extremities, along with numbness in the same distribution. Additionally, she displayed a decreased level of consciousness, deconjugate gaze, diplopia, and a positive Babinski sign. These clinical findings strongly suggested a stroke, a condition typically uncommon in a young patient without traditional stroke risk factors. Therefore, a comprehensive assessment of an underlying secondary cause was imperative.

The term ʽmalignant MCA infarctionʼ, first introduced in 1996, was initially defined as an infarction involving the complete MCA territory visible on CT scans within 48 h^4^. In this case, the patient’s CT scan revealed MCA occlusion, leading to malignant MCA syndrome. Consequently, she underwent a decompressive craniectomy.

A meta-analysis of population-based cohort studies reported a twofold increased risk of ischemic stroke, a threefold increased risk of intracerebral hemorrhage, and a fourfold increased risk of subarachnoid hemorrhage in individuals with SLE compared to the general population^5^. The increased risk of ischemic stroke in SLE patients can be attributed to various factors, including comorbid conditions that serve as stroke risk factors, accelerated atherosclerosis often seen in SLE, and systemic inflammation as a significant contributor to stroke risk^5^.

Two studies reported the effect of steroids on increasing the risk of stroke. The first one^6^ is a population-based longitudinal cohort study that mentioned that RA is a risk factor for ischemic stroke and steroids are a crucial factor in contributing to the risk of ischemic stroke among SLE patients. The second one, a nationwide population-based study^7^, reported a higher risk of SAH in SLE patients. Elderly SLE patients and those with a higher mean daily steroid dose and a history of platelet or red blood cell transfusion had a higher risk of developing SAH.

Another article published in 2012 about inflammation-induced thrombosis suggested three factors connecting chronic inflammation with thrombosis: hyperactive platelets, endothelium damage, or dysfunction, and increased procoagulant activity^8^.

However, inflammation-induced venous thrombosis may develop even in the absence of vessel wall damage. Proinflammatory cytokines and chemokines induce a procoagulant state in three ways: stimulating monocytes to produce tissue factor (TF), antithrombin III, and the protein C system.

CNS vasculitis, although extraordinarily rare, can contribute to cerebrovascular disease in SLE patients. Most cases of cerebrovascular disease in SLE are associated with a bland vasculopathy, often influenced by risk factors for atherosclerosis present in the non-SLE population^9^.

An article published in 2006 by the American College of Rheumatology about Platelet C4d as Highly Specific for Systemic Lupus Erythematosus^10^ Platelet C4d was detected in 18% of patients with SLE, being 100% specific for a diagnosis of SLE.

In addition, platelet C4d was significantly associated with positivity for lupus anticoagulant and anticardiolipin antibodies in the IgG. It was also significantly associated with SLE disease activity according to the SLE Disease Activity Index, low serum C4, an elevated ESR, and abnormal levels of C4d on erythrocytes.

The American College of Rheumatology (ACR) created standardized case definitions and diagnostic testing recommendations for 19 neuropsychiatric syndromes in SLE, including cerebrovascular disease^11^. Ischemic stroke is not uncommon in SLE patients, but it is rare to be the initial manifestation of the disease. It involves predominantly the posterior circulation, or ʽvertebrobasilar systemʼ. In our case, it affected the anterior circulation in the MCA.

A study published in the Republic of China in 2006 about neuropsychiatric manifestations in pediatric systemic lupus erythematosus: a 20-year study^12^. The study showed that 64 patients out of 185 patients (34.5%) developed neuropsychiatric symptoms. Fourteen patients (21.9%) had neuropsychiatric manifestations on the initial diagnosis of SLE in the forms of seizure, confusion, headache, polyneuropathy, CVA, depression, psychosis, demyelinating syndrome, and myopathy, with seizure being the most common among them all. Twenty-one patients (32.8%) developed NP symptoms within the first year of SLE, while 29 (45.3%) developed them after 1 year of SLE diagnosis. The CNS was involved in 96.2% of them, and the PNS was involved in 3.8%.

According to the EULAR/ACR criteria^13^, published in September 2019, the patient is diagnosed with SLE. She received 5 points for seizures and 6 points for anti-dsDNA, totaling 11 points and a positive ANA titer. She is not diagnosed with SLE according to the 1997 ACR criteria and is also not diagnosed according to the 2012 SLICC criteria.

The investigations used in the same study^12^ are as follows: The ANA level is elevated <1:1280 in 61% of the patients who were tested for it, 40% of NPSLE, and 68.9% of non-NPSLE. Our patient is ANA-positive. Anti-dsDNA is elevated in 73.7% of the patients who were tested for it, 32.6% of NPSLE, and 90.9% of non-NPSLE. Our patient is anti-dsDNA positive. The antiphospholipid antibody is elevated in 29.4% of the patients who were tested for it, 34.8% of NPSLE, and 25% of non-NPSLE; our patient is negative. The anticardiolipin antibody is elevated in 31.5% of the patients who were tested for it, 47.8% of NPSLE, and 19.4% of non-NPSLE. Our patient is negative. C3 and C4 levels are decreased in 90.7 and 80.6%, respectively, of the patients who were tested for them; C3 is decreased in 84.9% of NPSLE and 93.3% of non-NPSLE; C4 is decreased in 75% of NPSLE and 83.1% of non-NPSLE. Our patient has normal C3 and C4 levels. The patient also had an elevated ESR of 68 mm/h and a CRP of 8.6 mg/l.

A case report and review of the literature about ischemic stroke as the initial manifestation of SLE reported a case in addition to reviewing 10 cases^14^. The majority of the patients (7 of 11) had ischemic infarcts in the distribution of the vertebrobasilar system. The treatment choices were reported in only four of the cases. In all of them, immunosuppressant therapy (either with oral prednisolone or IV cyclophosphamide) was used in combination with oral anticoagulants or antiplatelet agents. For our patient, we also elected to utilize hydroxychloroquine, azathioprine, corticosteroids, aspirin, and enoxaparin.

EULAR published recommendations in 2010 for the management of systemic lupus erythematosus with neuropsychiatric manifestations^15^. The acute management of SLE stroke, or TIA, is similar to that in the general population, including aspirin ʽunless contraindicatedʼ. Secondary prevention includes tight control of cardiovascular risk factors, antiplatelet therapy, and carotid endarterectomy when indicated. Generalized lupus activity may be controlled with glucocorticoids and/or immunosuppressive therapy.

An article published in 2017^16^ recommends the following treatment regimens for stroke in SLE patients without antiphospholipid syndrome: hydroxychloroquine, aspirin (81–300 mg/days), if lupus activity: corticosteroids and immunosuppressants, aspirin, or clopidogrel.

Secondary prevention supports the control of generalized lupus activity with high doses of steroid (methylprednisolone) and immunosuppressants such as cyclophosphamide, azathioprine, mycophenolate mofetil, and in refractory cases, intravenous immunoglobulins, rituximab, and plasma exchange.

Another article^17^ shows that hydroxychloroquine acts on the control of disease activity and as an antithrombotic and antiatherosclerotic drug, with significant reductions in total cholesterol, low-density lipoprotein, and triglycerides.

## Conclusion

Diagnosing SLE always poses a challenge since the disease can resemble other conditions, particularly when the classic malar rash is absent and it presents in an unusual manner, such as an ischemic stroke. One of the most devastating forms of ischemic stroke is referred to as ʽmalignantʼ MCA infarction, estimated to account for ~10% of all ischemic strokes. This type of stroke carries an alarming fatality rate of up to 80% if left untreated. This case underscores a sudden and life-threatening manifestation of SLE, underscoring the critical significance of early recognition and tailored management, which can potentially alter the course of the disease.

## Ethical approval

This study is a case report, and our institution does not require ethical approval for such research, but they require obtaining the consent of the patient and the doctor supervising the case.

## Informed consent

Written informed consent was obtained from the patient’s parents/legal guardian for publication and any accompanying images. A copy of the written consent is available for review by the Editor-in-Chief of this journal on request.

## Consent for publication

All authors provide consent for publication.

## Sources of funding

There are no funding sources.

## Author contribution

All authors contributed in the work’s conception and design, paper writing, and article revision, and final revision and approval.

## Conflicts of interest disclosure

The authors declare that they have no competing interests.

## Research registration unique identifying number (UIN)

Our research study does not involve human subjects.

## Guarantor

Afnan .W. M. Jobran.

## Data availability statement

Not applicable.

## Provenance and peer review

Not commissioned, externally peer-reviewed.
